# Dietary inflammatory index and cardiovascular disease risk in Hispanic women from the Women’s Health Initiative

**DOI:** 10.1186/s12937-023-00838-9

**Published:** 2023-01-12

**Authors:** Monica D. Zuercher, Danielle J. Harvey, Margarita Santiago-Torres, Lauren E. Au, Nitin Shivappa, Aladdin H. Shadyab, Matthew Allison, Linda Snetselaar, Buyun Liu, John A. Robbins, James R. Hébert, Lorena Garcia

**Affiliations:** 1 University of California Davis, Davis, CA USA; 2 Division of Public Health Sciences, Fred Hutchinson Cancer Research Center, Seattle, WA USA; 3 Department of Epidemiology and Biostatistics and Cancer Prevention and Control Program, Arnold School of Public Health, University of South Carolina, Columbia, SC USA; 4 Herbert Wertheim School of Public Health and Human Longevity Science, University of California San Diego, La Jolla, CA USA; 5 Department of Family Medicine, University of California San Diego, La Jolla, CA USA; 6 Department of Epidemiology, University of Iowa, Iowa City, IA USA; 7 Medical Sciences 1C, UC Davis School of Medicine, Sacramento, USA

**Keywords:** Diet, Inflammation, Stroke, Coronary heart disease, Latinos

## Abstract

**Background:**

To evaluate the association between the dietary inflammatory index (DII^®^) and incident cardiovascular disease (CVD) in Hispanic women from the Women’s Health Initiative (WHI), and to determine if body mass index (BMI) interacted with the DII scores.

**Methods:**

Secondary analysis of baseline dietary data and long-term CVD outcomes among 3,469 postmenopausal women who self-identified as Hispanic enrolled in WHI. DII scores were calculated from self-administered food frequency questionnaires. The CVD outcomes included coronary heart disease (CHD) and stroke. Stratified Cox regression models were used to assess the relationship between DII scores and CVD in women with and without obesity. Models were adjusted for age, lifestyle risk factors, known risk factors, and neighborhood socioeconomic status.

**Results:**

The incidence of CHD was 3.4 and 2.8% for stroke after a median follow-up of 12.9 years. None of the DIIs were associated with CVD risk in this sample of Hispanic women. BMI interacted with the DII (*p* < 0.20) and stratified models showed that the associations between the DII and CVD were only significant in women with overweight (*p* < 0.05). In this group, higher DII scores were associated with a higher risk of CHD (HR 1.27; 95% CI: 1.08, 1.51) and a higher risk of stroke (HR 1.32; 95% CI: 1.07, 1.64).

**Conclusion:**

Among postmenopausal Hispanic women with overweight, greater adherence to pro-inflammatory diets was associated with higher risk of CVD. Additional research is needed to understand how to promote long-term heart-healthy dietary habits to reduce inflammation and prevent CVD in at-risk Hispanic women.

**Supplementary Information:**

The online version contains supplementary material available at 10.1186/s12937-023-00838-9.

## Background

Chronic inflammation plays a key role in the pathophysiology of cardiovascular disease (CVD), in the progression of atherosclerosis plaques, and the modulation of endothelial function [1, 2]. It is well known that diet contributes to the regulation of chronic inflammation through the modulation of the levels of inflammatory markers (i.e., cytokines, acute-phase proteins, soluble adhesion molecules, and cytokine receptors), as well as serum lipids and glucose [3–6]. Intakes of certain dietary components (i.e. red meat, high-fat dairy, refined grains, processed meat, sweets, desserts, and sugar-sweetened soft drinks) have been linked to higher levels of systemic inflammation, whereas intake of fruits, vegetables, whole grains, and dietary fiber have been linked with lower inflammation [3]. To facilitate research into the inflammatory effect of diet on health, information regarding the effect of individual dietary components over inflammation have been combined to create a tool, called the dietary inflammatory index (DII®) [4, 7].

The dietary inflammatory index (DII^®^) is a scoring algorithm that categorizes individuals’ diets on a continuum from maximally anti-inflammatory to maximally pro-inflammatory. This index was developed and validated to predict the concentration of six inflammatory biomarkers (interleukin (IL)-1β, IL-4, IL-6, IL-10, TNF-α, and C-reactive protein) based on the inflammatory score of a person’s diet [4, 7].

To our knowledge, there are only eight studies that have evaluated the association between the DII and CVD. The results from these studies were inconclusive and showed mixed results for women [2, 3, 8–13]. These inconsistencies emphasize the need for additional studies examining this association, especially in women since their clinical presentation of CVD differs from that of men. Moreover, none of the studies that have examined the relationship between DII and CVD were conducted in Hispanic individuals.

Notably, obesity is likely relevant to the pathologic process between dietary-related inflammation and chronic diseases because it can act as a confounder, as a mediator, and as an effect modifier [3, 12, 14]. There are strong correlations between pro-inflammatory diets and obesity and between obesity and chronic inflammation, which could result in a more detrimental effect of a pro-inflammatory diet in the development of disease when obesity is present [3, 14–16]. This complex interaction between obesity and dietary inflammation has been shown in different studies where the association between the DII or inflammatory factors and chronic diseases is stronger or only significant in participants with overweight or obesity but not in participants with normal weight [2, 6].

Given the paucity of data in Hispanic populations, this analysis presented below evaluated the association between the DII and CVD risk in a subset of self-reported Hispanic women from the Women’s Health Initiative. Analyses included adjustments for known risk factors for CVD such as genetic admixture, physical activity, acculturation, and diabetes at baseline, among others. Additionally, the interaction between the body mass index and the DII scores was evaluated.

## Methods

### Participants

The Women’s Health Initiative (WHI) enrolled 161,808 postmenopausal women across 40 WHI clinical centers nationwide between October 1, 1993, and December 31, 1998. Participants in the WHI study ranged in age from 50 to 79 years and were either randomized into three clinical trials (Hormone Therapy (HT) Trial, the Diet Modification (DM) Trial, and the Calcium and Vitamin D (CaD) Trial) or enrolled into an observational study (OS) [17]. The current study included 6,484 postmenopausal women who self-identified as Hispanic. Participants were excluded for a history of coronary heart disease (CHD) or stroke at baseline (*N* = 61), assignment to the treatment arm in the DM Trial (*N* = 751), energy consumption < 600 kcal/day or > 5000 kcal/day (*N* = 415), use of nonsteroidal anti-inflammatory drugs (*N* = 992), and missing genetic data (*N* = 857). After applying the exclusion criteria, the sample size for the analysis was 3,469 postmenopausal women (2,162 from the observational study and 1,307 from the clinical trials).

### Cardiovascular disease

The study outcomes included incident CHD and stroke. CHD was defined as hospitalized myocardial infarction, definite silent myocardial infarction, or death due to coronary disease. Stroke was defined as rapid onset of a persistent neurologic deficit attributed to an obstruction or rupture of the brain arterial system, lasting more than 24 h and without evidence for other cause. Only strokes requiring hospitalization were considered outcome events for WHI^10^. The CVD outcomes were adjudicated and ascertained by physician review of medical records, as previously described [18].

### Dietary Inflammatory Index (DII^®^)

Diet was evaluated with a standardized and validated self-administered food frequency questionnaire (FFQ) that was mailed to all participants at baseline [19]. The FFQ was developed to estimate the average daily consumption of 122 food items over the previous 3-month period and included information about the use of vitamin and mineral supplements. The estimation of the nutrient consumption of each participant was calculated using the University of Minnesota’s Nutrition Coordinating Center nutrient database, which is based on the U.S. Department of Agriculture Standard Reference Releases and manufacturer information [19].

The procedure used to calculate the DII scores from the FFQ responses from all subjects has been described elsewhere [4]. Briefly, the DII was created after an extensive literature review that identified 45 food parameters that were linked, with sufficiently robust literature, to six inflammatory biomarkers. Each whole food or nutrient received a food parameter-specific overall inflammatory effect score that was calculated based on the pro-inflammatory, anti-inflammatory, or null effect of that dietary component as reviewed in the scientific literature. Scoring also took into consideration the total number of articles published and the study design [4, 7].

Using the participants’ intake data as reported on the FFQ, a Z-score was calculated for each one of the available food parameters for each individual in the study based on the world average and standard deviation from a global composite dataset created for this purpose [4]. These Z-scores were converted to a proportion and then centered by doubling and subtracting 1. After this step, the centered proportion value for each food parameter was multiplied by the respective ‘overall food parameter-specific inflammatory effect score’ to obtain the ‘food parameter-specific DII score’ and finally, all the ‘food parameter-specific DII scores’ were added together to create the ‘overall DII score’ for an individual.

In the WHI FFQ, 32 of the 45 original DII components were available for inclusion in the overall DII score (Supplementary Table 1). The components ginger, turmeric, garlic, oregano, pepper, rosemary, eugenol, saffron, flavan-3-ol, flavones, flavonols, flavonones, and anthocyanidins that were included in the original DII calculation were not included in the WHI FFQ [6]. The WHI FFQ also included questions about the consumption of 15 nutritional supplements that are part of the DII components (iron, magnesium, niacin, riboflavin, selenium, thiamine, β-carotene, zinc, folic acid, and vitamins A, C, D, E, B6, and B12) [6]. Nevertheless, in our analysis the DII was calculated without considering the use of nutritional supplements.

### Covariates

The study covariates were determined using baseline data from both the observational study (OS) and clinical trial (CT) components of the WHI study. Demographic information included age, ethnicity, and preferred language. Neighborhood socioeconomic status (NSES) was evaluated using a standardized geocoding protocol, which linked individual WHI participant addresses to the year 2000 U.S. Census Federal Information Processing Standards (FIPS) codes and tract-level socioeconomic data. A summary measure of each participant's neighborhood socioeconomic environment was estimated from the tract-level data using six variables representing several dimensions of wealth and income [20]. Higher NSES scores represent a higher neighborhood socioeconomic status.

Risk factors for CVD were also included: physical activity, smoking status, acculturation, alcohol intake, body mass index (BMI), genetic admixture, and other chronic diseases. Physical activity was evaluated with a validated physical activity questionnaire, and it was included in the model as the total minutes of recreational physical activity per week, including walking, mild, moderate, and strenuous physical activity [21]. Smoking status was determined as never or past/current smoker. Language preference (English or Spanish) was used as a proxy measure for acculturation status. Alcohol intake in grams per day was estimated using a food frequency questionnaire [19]. For BMI, weight was measured to the nearest 0.1 kg on a balance beam scale. Height was measured to the nearest 0.1 cm using a wall-mounted Harpenden stadiometer. BMI was calculated as weight (kg) divided by the square of measured height (m^2^) [22]. Hypertension was defined as systolic pressure ≥ 130 mm Hg or diastolic ≥ 80 mm Hg or self-reported hypertension with the use of antihypertensive medication [23]. Diagnosis of diabetes at baseline was obtained from the medical history questionnaire in response to the question “Did a doctor ever say that you had sugar diabetes or high blood sugar when you were not pregnant?” [24]. Hypercholesterolemia was defined by self-report at baseline and then by use of lipid-modulating medication [23]. Obesity was defined as a BMI ≥ 30 km/m^2^.

Genetic ancestry is an important and often ignored factor that may affect the way that ethnic groups respond to dietary patterns and that can alter the relationship between diet-associated inflammation and risk of metabolic diseases [25–29]. This study includes Amerindian ancestry as a covariate to account for the genetic diversity of Hispanic women when evaluating the relationship between dietary patterns and CVD. Genetic admixture was calculated using a marker set of 92 ancestry informative markers that demonstrated large differences in allele frequency between ancestral populations from Europe, sub-Saharan Africa, and the Americas (> 45%) [30, 31]. Genotyping was performed using the TaqMan OpenArrays system (Life Technologies/Applied Biosystems, Foster City, CA, USA) [30]. Admixture proportions were determined using the Bayesian clustering algorithms implemented in the program STRUCTURE v2.1 [31, 32].

### Statistical analysis

Two-sample t-tests were applied to compare the mean differences of each continuous variable between participants with and without CVD. Chi-square tests were used to compare the two groups on categorical variables. Separate Cox regression models were fit to examine the association between DII and CHD/stroke. The analyses were restricted only to follow-up events and time to CHD/stroke occurrence were the outcomes of interest. CHD and stroke status were used as dichotomous traits (0 = no, 1 = yes) as the indicator variable for failure/censorship. The survival time for participants who did not develop the CVD outcome of interest was defined as the days from enrollment to the end of follow-up (the follow-up time for CVD events in this analysis includes data until September 2018). Hazard ratios (HRs) and 95% confidence intervals (CI’s) are presented for each model. Cox regression models were conducted with and without adjusting for age at entry, lifestyle-related risk factors (smoking, alcohol intake, physical activity, and acculturation), known CVD risk factors (diabetes, hypertension, hypercholesterolemia, BMI, and Amerindian ancestry), and neighborhood socioeconomic status (NSES). These covariates were serially added to the model and the final model included all covariates. Multiple imputation with the fully conditional specification method was used to estimate missing values of the variable NSES (*N* = 273 which represents 7.9% of the observations) and physical activity (*N* = 167 which represents 4.8% of the observations) assuming that data were missing at random. A sensitivity analysis excluding women in the control arm of the dietary modification trial was performed. Finally, the interaction between BMI and the DII scores was evaluated by adding interaction terms between BMI and the DII scores in the final models. Stratified models for women in the different BMI categories (normal weight BMI 18.5–24.5 kg/m^2^, overweight BMI 25.0–29.9 kg/m^2^ and obesity BMI > 30 kg/m^2^) were fitted if needed. Analyses were performed using SAS software version 9.4 (SAS Institute, Cary, NC USA). All statistical tests were two-sided and *p* ≤ 0.05 was considered statistically significant.

## Results

The secondary analysis included 3,469 postmenopausal women who self-identified as Hispanic. Of these, 49.0% were of Mexican origin, 11.6% of Puerto Rican origin, 8.2% of Cuban origin, and 26.1% other Hispanic/Latina. The mean DII score was 1.9 ± 2.3 (min: -5.3, max: 5.7). After a median follow-up of 12.9 years (range, 0.1–24.0 years) the incidence of CHD was 3.4% and the incidence of stroke was 2.8%.

Women in the highest DII tertile (corresponding to more inflammatory diets) had higher values of Amerindian ancestry proportion, than women in the lowest tertile (corresponding to less inflammatory diets) (*p* < 0.05) (Table 1). Women in the highest tertile had lower values of neighborhood socioeconomic level, physical activity, obesity, energy and nutrients intake, and alcohol consumption than women in tertile 1 (*p* < 0.05). Finally, there were no statistically significant differences in BMI, age, acculturation (use of English as their preferred language), allocation in the hormone treatment arm, use of nutritional supplements, and the prevalence of smoking, hypertension, hypercholesterolemia, or diabetes between these women (*p* > 0.05).

**Table 1:** Comparison of descriptive characteristics of women with less inflammatory diets (DII score tertile 1) vs. women with more inflammatory diets (DII score tertile 3)

Variable	Tertile 1^b^ (*n* = 1156)		Tertile 3^b^ (*n* = 1156)		*P*-Value
	Mean	SD	Mean	SD	
Age (years)	60.3	6.9	60.1	6.9	0.33
Neighborhood SES^a^ (score)	69.4	10.2	68.0	10.4	0.002
Physical Activity (hr/wk)	183.2	192.7	143.5	171.0	< 0.0001
Alcohol intake (g/day)	1.5	3.8	0.9	2.6	< 0.0001
BMI^a^ (kg/m^2^)	28.9	5.6	28.5	5.7	0.08
AMI^a^ genetic ancestry (proportion)	0.26	0.2	0.28	0.2	0.003
Energy intake (kcal/d)	2254.5	818.2	1049.7	322.2	< 0.0001
Carbohydrates (g/day)	284.8	95.5	130.8	45.7	< 0.0001
Total Fat (g/day)	83.0	44.1	40.2	17.8	< 0.0001
Saturated Fat (g/day)	27.1	15.5	13.1	6.5	< 0.0001
Trans Fat (g/day)	5.2	3.5	2.6	1.6	< 0.0001
Cholesterol (mg/day)	310.4	184.8	157.1	86.3	< 0.0001
Protein (g/day)	95.3	36.4	41.3	13.9	< 0.0001
Sugar (g/day)	129.2	59.4	62.4	32.8	< 0.0001
Dietary Fiber (g/day)	22.9	7.5	9.1	3.2	< 0.0001
**Variable**	**n**	**(%)**	**n**	**(%)**	*P*-Value
Obesity (yes)	417	36.6	371	32.5	0.04
Smoking (yes)	394	34.9	412	36.3	0.47
Hypertension (yes)	659	57.0	674	58.3	0.53
Hypercholesterolemia (yes)	168	15.6	165	15.4	0.87
Diabetes (yes)	93	8.1	94	8.1	0.94
Preferred language (English)	871	75.4	866	74.9	0.81
Nutritional supplements use (yes)	606	52.4	563	48.7	0.07
Hormone treatment arm (yes)	244	21.1	275	23.8	0.12

^a^*DII* Dietary inflammatory index, *SES* Socioeconomic status, *BMI* Body mass index, *AMI* Amerindian

^b^The DII scores in women in the highest tertile ranged from 3.23 to 5.75 and the DII scores in women in the lowest tertile ranged from -5.29 to 1.06

The DII was associated with a higher risk of CHD when adjusting for age only (HR 1.09; 95% CI: 1.003, 1.19 for a 1-unit increasement of the DII score) but the association was no longer statistically significant when covariates were added to the model (*p* > 0.05) (Table 2). The DII was not statistically significantly associated with risk of stroke (Table 3) (*p* > 0.05). Sensitivity analyses showed that adding a variable indicating if a woman was allocated or not in the hormone treatment arm or in the control arm of the dietary modification trial did not change the hazard ratios significantly (% of change < 1%) (data not shown). Exploratory analysis that included the use of nutritional supplements in the calculation of the DII showed that the DII with supplements was not associated with risk of CVD (data not shown).

**Table 2:** Association of the dietary inflammatory index and coronary heart disease in Hispanic women

Model	Variables	HR	95% CI	*P*-Value
All women				
1	Model 1	1.09	1.003, 1.19	0.04
2	Model 1 + lifestyle-related risk factors	1.08	0.99, 1.18	0.08
3	Model 2 + known risk factors	1.08	0.99, 1.17	0.10
4	Model 3 + socioeconomic covariates	1.07	0.98, 1.17	0.13
Women with normal weight				
1	Model 1	1.02	0.85, 1.23	0.80
2	Model 1 + lifestyle-related risk factors	1.04	0.86, 1.25	0.69
3	Model 2 + known risk factors	1.03	0.82, 1.25	0.78
4	Model 3 + socioeconomic covariates	1.03	0.82, 1.25	0.78
Women with overweight				
1	Model 1	1.32	1.12, 1.56	0.001
2	Model 1 + lifestyle-related risk factors	1.28	1.09, 1.51	0.003
3	Model 2 + known risk factors	1.28	1.08, 1.51	0.004
4	Model 3 + socioeconomic covariates	1.27	1.08, 1.51	0.005
Women with obesity				
1	Model 1	0.97	0.86, 1.10	0.64
2	Model 1 + lifestyle-related risk factors	0.97	0.85, 1.10	0.60
3	Model 2 + known risk factors	0.97	0.85, 1.11	0.66
4	Model 3 + socioeconomic covariates	0.96	0.84, 1.09	0.53

^*^Model 1: with age at baseline. Model 2: with age at baseline and lifestyle-related risk factors. Model 3: with age at baseline, lifestyle-related risk factors, and known risk factors. Model 4: with age at baseline, lifestyle-related risk factors, known risk factors, and socioeconomic covariates

^†^Lifestyle-related risk factors: smoking, alcohol intake, physical activity, and acculturation; known risk factors: diabetes, hypertension, hypercholesterolemia, body mass index, and Amerindian ancestry; Socioeconomic covariates: neighborhood socioeconomic status

^‡^Sample size 911 women with normal weight (BMI 18.5—24.9 kg/m2), 1332 women with overweight (BMI 25.0—29.9 kg/m2) and 1150 women with obesity (BMI ≥ 30 kg/m^2^)

**Table 3:** Association of the dietary inflammatory index and stroke in Hispanic women

Model	Variables	HR	95% CI	*P*-Value
All women				
1	Model 1	1.05	0.96, 1.16	0.27
2	Model 1 + lifestyle-related risk factors	1.03	0.94, 1.13	0.53
3	Model 2 + known risk factors	1.03	0.94, 1.13	0.56
4	Model 3 + socioeconomic covariates	1.03	0.93, 1.13	0.59
Women with normal weight				
1	Model 1	1.00	0.84, 1.19	0.99
2	Model 1 + lifestyle-related risk factors	0.98	0.82, 1.17	0.83
3	Model 2 + known risk factors	0.98	0.82, 1.18	0.86
4	Model 3 + socioeconomic covariates	0.98	0.82, 1.18	0.85
Women with overweight				
1	Model 1	1.36	1.10, 1.67	0.004
2	Model 1 + lifestyle-related risk factors	1.36	1.10, 1.69	0.005
3	Model 2 + known risk factors	1.34	1.08, 1.66	0.008
4	Model 3 + socioeconomic covariates	1.32	1.07, 1.64	0.01
Women with obesity				
1	Model 1	0.94	0.83, 1.07	0.36
2	Model 1 + lifestyle-related risk factors	0.91	0.79, 1.04	0.16
3	Model 2 + known risk factors	0.90	0.79, 1.04	0.15
4	Model 3 + socioeconomic covariates	0.91	0.79, 1.05	0.19

^*^Model 1: with age at baseline. Model 2: with age at baseline and lifestyle-related risk factors. Model 3: with age at baseline, lifestyle-related risk factors, and known risk factors. Model 4: with age at baseline, lifestyle-related risk factors, known risk factors, and socioeconomic covariates

^†^Lifestyle-related risk factors: smoking, alcohol intake, physical activity, and acculturation; known risk factors: diabetes, hypertension, hypercholesterolemia, body mass index, and Amerindian ancestry; Socioeconomic covariates: neighborhood socioeconomic status

^‡^Sample size 900 women with normal weight (BMI 18.5—24.9 kg/m2), 1310 women with overweight (BMI 25.0—29.9 kg/m2) and 1122 women with obesity (BMI ≥ 30 kg/m2

The interaction terms between BMI and the DII were statistically significant in the fully adjusted models for both CVD outcomes (p for interaction terms < 0.20) (Supplementary Table 2). When stratified by the different BMI categories, the significance and the magnitude of the associations were different between women in the different BMI categories (Tables 2 and 3). Specifically, the associations were not statistically significant in women with normal weight or obesity but were statistically significant for women with overweight. In women with overweight, the DII was associated with a higher risk of CHD even after adjusting for covariates (HR 1.27; 95% CI: 1.08, 1.51) (Table 2) and with a higher risk of stroke (HR 1.32; 95% CI: 1.07, 1.64) (Table 3). (The characteristics of the participants by the different BMI categories are shown in Supplementary Table 3).

## Discussion

Overall, we found that DII was not statistically significantly associated with CVD risk among postmenopausal Hispanic women. This is consistent with previous reports where the association between the DII and CVD is not statistically significant or is only significant in men but not in women [3, 12]. Other studies have found significant associations between the DII and CVD, but it is important to mention that the differences in the results could be affected by differences in the characteristics of the participants (i.e., gender distribution, age and country of origin), differences in the CVD outcome used (i.e., use of composed outcomes and CVD indicators like mortality or sub-clinical CVD conditions as the outcome), and methodological differences (i.e., cross-sectional designs and differences in the food items included in the FFQs used to measure dietary intake) [2, 8–11, 13].

Body mass index appeared to modify the association between DII and CVD. That is, among women with normal weight and obesity, there was no statistically significant association between the DII and CVD outcomes. However, in women with overweight, the DII was associated with a higher risk of CHD and stroke. Similar results were found in the Supplèmentation en Vitamines et Minèraux AntioXydants (SU.VI.MAX) cohort study that enrolled 7,743 French adults (women aged 35–60 years and men aged 45–60 years) [10]. Results from this study showed that a higher DII score was associated with a higher risk of myocardial infarction (HR for quartile 4 vs. quartile 1 = 2.24; 95% CI 1.08–4.67).

The results from our study partially support our hypothesis that the association between DII scores and CVD would be stronger in participants with overweight or obesity but not in participants with normal weight. Other studies have found that the association between the DII or inflammatory factors and chronic diseases were stronger or only significant in participants with overweight/obesity but not in participants with normal weight [2, 6]. The differences in the findings could be explained by differences in the characteristics of the participants of the studies because previous studies involved mostly White populations, so it is possible that this association in participants with obesity is different in Hispanics. Another potential explanation may be that obesity is associated with chronic inflammation and the inflammatory “potential” of the diet might have been too small to counter the systemic pro-inflammatory effect of obesity [i.e. ceiling effect] [33, 34]. A pilot clinical controlled trial that evaluated whether antioxidants from either food or supplement sources lowered the blood concentration of inflammatory factors in adults with at least one elevated risk factor for CVD (overweight/obesity, hypertension, or elevated blood lipids) found that there were no beneficial effects on the inflammatory markers investigated in response to antioxidants from foods or supplements [35]. This suggests that the inflammatory potential of the diet may play a smaller role in the development of cardiovascular disease than other risk factors that have a stronger effect on systemic inflammation in the setting of CVD risk factors such as obesity.

In this study, DII with supplements was not associated with risk of CVD. Around 51% of the women in this study reported using nutritional supplements (data not shown). Because 13 of the 15 nutritional supplements included in the WHI FFQ have been attributed with anti-inflammatory properties, this caused that the DII scores with nutritional supplements were more skewed towards anti-inflammatory scores than the DII that do not included nutritional supplements. Moreover, the effect of nutritional supplements on chronic inflammation remains unclear and this may also explain the lack of association between the indexes that include nutritional supplements compared to those that do not include them in their calculation. The existing nutritional literature shows mixed results with beneficial, null, or detrimental effects attributed to the use of nutritional supplements and most of the studies have methodological limitations that need to be considered when interpreting the results [35–38].

The strengths of this study included a large study population with women from diverse Hispanic heritage backgrounds and the longitudinal prospective study design. In addition, the WHI FFQ was designed to reflect regional and ethnic eating patterns of the United States. Twelve foods were added to reflect Hispanic eating patterns and there was a large set of physical, biological, and social covariates available in the WHI database to reduce the amount of residual confounding of the models. Moreover, the DII has been validated to predict concentrations of inflammatory markers in the WHI [6].

The limitations of this study include the use of only baseline dietary information that did not allow evaluation of inflammation changes in the diet over time. A previous study conducted in the whole sample of the WHI study showed that the DII score decreased slightly over the initial 3 years of WHI enrollment, indicating a small shift toward a less inflammatory diet during the study period [39]. By using only dietary information at one point in time, we were unable to adjust dietary changes during the follow-up time. Other limitations include dietary measurement errors associated with self-report of diet, such as the potential for social desirability, recall bias, and the missing 13 anti-inflammatory components from the original DII calculation [40]. It is well known that self-report of dietary energy and protein intakes has been systematically and differentially underreported in the WHI, and this underreporting is greater in Hispanic American women than in White American women [41]. This underreporting of energy and protein can bias the DII scores to lower inflammatory values. Moreover, the 3-month recall period of the FFQ is relatively short so it might not represent the usual dietary intake of the participant thought the whole year and it might be susceptible to seasonal deviations on eating patterns depending on the month when it is filled out. The missing 13 anti-inflammatory components were not estimated in the WHI study because this information was not included in the FFQ would have a minimal impact on overall DII scores because many are consumed in small quantities. Evidence of this small impact was demonstrated in the study conducted by Tabung et al. (2015) where the DII was significantly associated with inflammatory biomarkers in the WHI.

## Conclusion

Pro-inflammatory diets (greater DII scores) were associated with a higher risk of CHD and a trend to increase the risk of stroke only in Hispanic women with overweight. This finding suggests that postmenopausal women with overweight might benefit more from anti-inflammatory diets in the prevention of cardiovascular disease than postmenopausal women with obesity. Further study is needed to determine the pathophysiological link between diet, inflammation, and cardiovascular disease in Hispanic women. Recommendations for future research include the enrollment of participants from different Hispanic heritage groups, a FFQ designed to collect information about the consumption of the 45 DII components, the use of biomarkers, and repeated measures in the collection of dietary information.

## Supplementary Information

**Additional file 1:** **Supplementary Table 1.** Components of the Dietary Inflammatory Index available in the WHI food frequency questionnaire*. **Supplementary Table 2.** Test for interaction between the dietary inflammatory index and obesity. **Supplementary Table 3.** Comparison of descriptive characteristics of women with different BMI categories.

## Data Availability

The datasets generated during and/or analyzed during the current study are not publicly available due to the data belongs to the Women’s Health Initiative and authors are under a data use agreement that do not allows them to share the data.
