# Supplementary material for: Dietary inflammatory index and cardiovascular disease risk in Hispanic women from the Women’s Health Initiative
Source: Nutr J. 2023 Jan 12;22:5. doi: 10.1186/s12937-023-00838-9 (PMC9835220; doi:10.1186/s12937-023-00838-9)
Supplement: Supplementary file 1 — Additional file 1: Supplementary Table 1. Components of the Dietary Inflammatory Index available in the WHI food frequency questionnaire*. Supplementary Table 2. Test for interaction between the dietary inflammatory index and obesity. Supplementary Table 3. Comparison of descriptive characteristics of women with different BMI categories. [file 12937_2023_838_MOESM1_ESM.docx]

Supplementary Table 1. Components of the Dietary Inflammatory Index available in the WHI food frequency questionnaire*

|  | DII component |  | DII component |
| --- | --- | --- | --- |
| 1 | Alcohol, g | 17 | Omega 6, g |
| 2 | Vitamin B12, ug | 18 | Onion, g |
| 3 | Vitamin B6, mg | 19 | Protein, g |
| 4 | Beta Carotene, ug | 20 | PUFA, g |
| 5 | Caffeine, g | 21 | Riboflavin, mg |
| 6 | Carbohydrate, g | 22 | Saturated Fat, g |
| 7 | Cholesterol, mg | 23 | Selenium, mg |
| 8 | Energy, kcal | 24 | Thiamin, mg |
| 9 | Total Fat, g | 25 | Trans Fat, g |
| 10 | Fiber, g | 26 | Vitamin A, ug |
| 11 | Folic Acid, mg | 27 | Vitamin C, mg |
| 12 | Iron, mg | 28 | Vitamin D, ug |
| 13 | Magnesium, mg | 29 | Vitamin E, mg |
| 14 | MUFA, g | 30 | Zinc, mg |
| 15 | Niacin, mg | 31 | Green tea/Black tea, g |
| 16 | Omega 3, g | 32 | Isoflavones, mg |
|  |  |  |  |

*DII, Dietary inflammatory index. WHI, Women’s Health Initiative.

Supplementary Table 2. Test for interaction between the dietary inflammatory index and obesity.

| Outcome | Variables | P-Value of interaction term |
| --- | --- | --- |
| CHD | Model 4+ DII*BMI | 0.11 |
| Stroke | Model 4+ DII*BMI | 0.08 |

*CHD, coronary heart disease. DII, dietary inflammatory index.

^†^Model 4 is adjusted by age at baseline, lifestyle-related risk factors, known risk factors and socioeconomic covariates.

Supplementary Table 3. Comparison of descriptive characteristics of women with different BMI categories.

| Variable | Normal weight (n=911) | | Overweight (n=1332) | | Obesity (n=1177) | | P-Value^†^ |
| --- | --- | --- | --- | --- | --- | --- | --- |
|  | Mean | SD | Mean | SD | Mean | SD |  |
| Age (years) | 60.7 | 7.2 | 60.4 | 6.8 | 59.6 | 6.5 | 0.001^b,c^ |
| Neighborhood SES* (score) | 71.1 | 10.0 | 68.8 | 10.4 | 67.2 | 9.9 | <0.0001^a,b,c^ |
| Physical Activity (hr/wk) | 198.1 | 199.2 | 173.1 | 182.6 | 132.5 | 170.4 | <0.0001^a,b,c^ |
| Alcohol intake (g/day) | 1.6 | 3.9 | 1.2 | 3.0 | 1.2 | 4.5 | 0.01^a,b^ |
| AMI* genetic ancestry (proportion) | 0.2 | 0.2 | 0.3 | 0.2 | 0.3 | 0.2 | 0.003 ^a,b^ |
| Energy intake (kcal/d) | 1463.0 | 647.7 | 1588.3 | 760.3 | 1725.9 | 819.6 | <0.0001 ^a,b,c^ |
| Carbohydrates (g/day) | 191.5 | 82.9 | 200.7 | 96.5 | 209.6 | 98.7 | <0.0001 ^b,c^ |
| Total Fat (g/day) | 51.1 | 30.8 | 59.0 | 35.0 | 67.4 | 39.7 | <0.0001 ^a,b,c^ |
| Saturated Fat (g/day) | 16.7 | 10.7 | 19.3 | 12.1 | 22.0 | 13.7 | <0.0001 ^a,b,c^ |
| Trans Fat (g/day) | 3.1 | 2.4 | 3.7 | 2.7 | 4.3 | 3.1 | <0.0001 ^a,b,c^ |
| Cholesterol (mg/day) | 190.7 | 124.2 | 221.6 | 142.4 | 259.6 | 167.8 | <0.0001 ^a,b,c^ |
| Protein (g/day) | 59.5 | 27.9 | 65.3 | 33.8 | 71.6 | 36.8 | <0.0001 ^a,b,c^ |
| Sugar (g/day) | 89.0 | 45.2 | 93.4 | 54.1 | 96.1 | 57.9 | 0.01^b^ |
| Dietary Fiber (g/day) | 16.3 | 7.5 | 15.1 | 7.9 | 15.4 | 7.7 | 0.72 |
| Variable | n | (%) | n | (%) | n | (%) | P-Value |
| Smoking (yes) | 313 | 35.0 | 481 | 36.9 | 430 | 37.2 | 0.54 |
| Hypertension (yes) | 398 | 43.7 | 737 | 55.3 | 799 | 67.9 | <0.0001^a,b,c^ |
| Hypercholesterolemia (yes) | 129 | 15.1 | 188 | 15.3 | 171 | 15.6 | 0.95 |
| Diabetes (yes) | 36 | 4.0 | 92 | 6.9 | 160 | 13.6 | <0.0001^a,b,c^ |
| Preferred language (English) | 729 | 80.0 | 966 | 72.5 | 890 | 75.6 | 0.0003^a,b^ |
| Nutritional supplements use (yes) | 565 | 62.0 | 685 | 51.4 | 510 | 43.3 | <0.0001^a,b,c^ |
| Hormone treatment arm (yes) | 152 | 16.7 | 300 | 22.5 | 320 | 27.2 | <0.0001^a,b,c^ |

^†^P-values represent the overall effect of BMI categories. Statistical significance for pairwise comparisons is indicated as follow: ^a^differences between normal weight and overweight, ^b^differences between normal weight and obesity, and ^c^differences between overweight and obesity.

* DII, dietary inflammatory index. SES, socioeconomic status. BMI, body mass index. AMI, Amerindian.
